# Supplementary material for: Heat adaptation of phage T7 under an extended genetic code
Source: Virus Evol. 2021 Dec 1;7(2):veab100. doi: 10.1093/ve/veab100 (PMC8923235; doi:10.1093/ve/veab100)
Supplement: veab100_Supp [file veab100_supp.zip › Sequencing_Analysis.zip!/Sequencing_Analysis/A1/evidence/RA_28.html]

BRESEQ :: Evidence


| Read alignment evidence... | | | | | | | | | | | |
| --- | --- | --- | --- | --- | --- | --- | --- | --- | --- | --- | --- |
|  | seq id | position | | ref | new | freq | score (cons/poly) | reads | annotation | genes | product |
| \* | NC\_001604.1 | 26,148 | 0 | A | G | 62.8% | 2456.9 / inf | 2705 | intergenic (–/–) | *–/–* | –/– |
| Reads supporting (aligned to +/- strand):  **ref** base A (479/510);  **new** base G (770/902);  **total** (1269/1436) | | | | | | | | | | | |
| Fisher's exact test for biased strand distribution *p*-value = 2.44e-01 | | | | | | | | | | | |
| Kolmogorov-Smirnov test that lower quality scores support variant *p*-value = 3.05e-01 | | | | | | | | | | | |

Only 100 of 2743 total aligned reads displayed.  

`CACCAACCCTTGGCCTTCTTTTGTTGGTTCAAGTATTAACGATGTGTTCTTCTTCCGTAACCGCTTAGGATTCCTTAGTGGGGAGAACATCATATTGAGTCGTACAGCCAAATACTTCAACTTCTACCCTGCGTCCATTGCGAACCTTAGTGATGACGACCCTATAGACGTAGCTGTGAGTACCAACCGAATAGCAATCCTTAAGTACGCCGTTCCGTTCTCAGAAGAGTTACTCATCTGGTCCGATGAAGCACAATTCGTCCTGACTGCCTCGGGTACTCTCACATCTAAGTCGGTTGAGTTGAACCTAACGACCCAGTTTGACGTACAGGACCGAGCGAGACCTTTTGGGATTGGGCGTAATGTCTACTTTGCTAGTCCGAGGTCCAGCTTCACGTCCATCCACAGGTACTACGCTGTGCAGGATGTCAGTTCCGTTAAGAATGCTGAGGACATTACATCACACGTTCCTAACTACATCCCTAATGGTGTGTTCAGTATTTGCGGAAGTGGTACGGAAAACTTCTGTTCGGTACTATCTCACGGGGACCCTAGTAAAATCTTCATGTACAAATTCCTGTACCTGAACGAAGAGTTAA  >  NC_001604.1/25849‑26447`  

                                                                                                                                                                                                                                                                                                           `|`                                                                                                                                                                                                                                                                                                             
      `cccTTGGCCTTCTTTTGTTGGTTCAAGTATTAACGATGTGTTCTTCTTCCGTAACCGCTTTGGATTCCTTAGTGGGGAGAACATCATATTGAGTCGTACAGCCAAATACTTCAACTTCTACCCTGCGTCCATTGCGAACCTTAGTGATGACGCCCCTATAGACGTATCTGTGAGTACCAACCGAATAGCAATCCTTAAGTACGCCGTTCCGTTTTCAGTAGAGTTACTCATCTGGTCCGATGAAGCACAATTCGTCCTGACTGCCTCGGGTACTCTCCCATCTAAGTCGGTTGGGTTGAAc                                                                                                                                                                                                                                                                                                      >  1:445097/1‑301 (MQ=255)`  
      `cccTTGGCCTTCTTTTGTTGGTTCAAGTATTAACGATGTGTTCTTCTTCCGTAACCGCTTAGGATTCCTTAGTGGGGAGAACGTCATATTGCGTCGTACAGCCAAATACTTCAACTTCTACCCTGCGTCCATTGCGAACCTTAGTGATGACGACCCTATAGACGTAGCTGTGAGTACCAACCGAATACCAATCCTTAAGTACGCCGTTCCGTTCTCAGAAGAGTTACTCATCTGGTCCGATGAAGCCAAATTCCTCCTGCCTGCCTCGGGTTATCTCACATCTTAATCGGTTGGGTTCAAc                                                                                                                                                                                                                                                                                                      >  1:407110/1‑301 (MQ=255)`  
      `cccTTGGCCTTCTTTTGTTGGTTCAAGTATTAACGATGTGTTCTTCTTCCGTAACCGCTTAGGATTCCTTAGTGGGGAGAACATCATATTGAGTCGTACAGCCAAATACTTCAACTTCTACCCTGCGTCCATTGCGAACCTTAGTGATGACGACCCTATAGACGTAGCTGTGAGTACCAACCGAATAGCAATCCTTAAGTACGCCGTTCCGTTCTCAGAAGAGTTACTCATCTGGTCCGATGAAGCACAATTCGTCCTGACTGCCTCGGGTACTCTCACATCTAAGTCGGTTGGGTTGAAc                                                                                                                                                                                                                                                                                                      <  1:377179/301‑1 (MQ=255)`  
      `cccTTGGCCTTCTTTGGTTGGTTCAAGTATTAACGATGTGTTCTTCTTCCGTAACCGCTTAGGATTCCTTAGTGGGGAGAACATCATATTGAGTGGTACAGCCAAATACTTGAACTTCTACCCTGCGTCCATTGCGAACCTTAGTGATGACGACCCTATAGACGTAGCTGTGAGTACCAACCGAATAGCAATCCTTAAGTCCGCCGTTCCGTTCTCAGAAGAGTTACTCATCTGGTCCGATGAAGCACAATTCGTCCTGACTGCCTCGGGTACTCTCACATCTAAGTCGGTTGAGTTGAAc                                                                                                                                                                                                                                                                                                      <  1:282118/301‑1 (MQ=255)`  
       `ccTTGGCCTTCTTTTGTTGGTTCAAGTATTAACGATGTGTTCTTCTTCCGTAACCGCTTAGGATTCCTTAGTGGGGATAACATCATATTGAGTCGTACAGCCAAATACTTCAACTTCTACCCGGCGTCCATTGCGAACCTTAGTGATGACGACCCTATAGACGTAGCTGTGAGTACCAACCGAATAGCAATCCTTAAGTACGCCGTTCCGTTCTCAGAAGAGTTACTCATCTGGTCCGATGAAGCACAATTCGTCCTGACTGCCTCGGGTACTCTCACATCTAAGTCGGTTGGGTTGAAcc                                                                                                                                                                                                                                                                                                     <  1:325286/301‑1 (MQ=255)`  
           `gagatgtgtataagagacagaGTATTAACGATGTGTTCTTCTTCCGTAACCGCTTAGGATTCCTTAGTGGGGAGAACATCATATTGAGTCGTACAGCCAAATACTTCAACTTCTACCCTGCGTCCATTGCGAACCTTAGTGATCACGACCCTATAGACGTAGCTGTGAGTACCAACCGAATAGCAATCCTTAAGTACGCCGTTCCGTTCTCAGAAGAGTTACTCATCTGGTCCGATGAAGCACAATTCGTCCTGACTGCCTCGGGTACTCTCACATCTAAGTCGGTTGGGTTGAACCTAAc                                                                                                                                                                                                                                                                                                 <  1:260519/281‑1 (MQ=255)`  
              `cagcttTTGTTGGTTCAAGTATTAACGATGTGTTCTTCTTCCGTAACCGCTTAGGATTCCTTAGTGGGGAGAACATCATATTGAGTCGTACAGCCAAATACTTCAACTTCTACCCTGCGTCCATTGCGAACCTTATTGATGACGACCCTATAGACGTAGCTGTGAGTACCAACCGAATAGCAATCCTTAAGTACGCCGTTCCGTTCTCAGAAGAGTTACTCATCTGGTCCGATGAAGCACAATTCGTCCTGACTGCCTCGGGTACTCTCACATATAAGTCGGTTGGGTTGAACCTAACGAc                                                                                                                                                                                                                                                                                              <  1:6036/298‑1 (MQ=255)`  
                   `tttGTTGGTTCAATTATTAACGATGTGTTCTTCTTCCGTAACCGCTTAGGATTCCTTAGTGGGGAGAACATCATATTGAGTCGTACAGCCAAATACTTCAACTTCTATCCTGCGTCCATTGCGAACCTTAGTGATGACGACCCTATAGACGTAGCTGTGAGTACCAACCGAATAGCAATCCTTAAGTACGCCGTTCCGTTCTCAGAAGAGTTACTCATCTGGTCCGATGAAGCACAATTCGTCCTGACTGCCTCGGGTACTCTCACATCTAAGTCGGTTGGGTTGAACCTAACGACCCAGt                                                                                                                                                                                                                                                                                         <  1:329159/301‑1 (MQ=255)`  
                         `agacagaGTATTAACGATGTGTTCTTCTTCCGTAACCGCTTAGGATTCCTTAGTGGGGAGAACATCATATTGAGTCGTACAGCCAAATACTTCAACTTCTACCCTGCATCCATTGCGAACCTTAGTGATGACGACCCTATAGACGTAGCTGTGAGTACCAACCGAATAGCAATCCTTAAGTACGCCGTTCCGTTCTCAGAAGAGTTACTCATCTGGTCCGATGAAGCACAATTCGTCCTGACTGCCTCGGGTACTCTCACATCTAAGTCGGTTGGGTTGAACCTAACGACCCAGTTTGACg                                                                                                                                                                                                                                                                                   <  1:320584/295‑1 (MQ=255)`  
                            `tCAAGTATTAAAGATGTGTACTTCTTCCGTAACCGCGTAGGATTCCTTAGTGGGGAGAACATCATATTGAGTCGTACAGCCAAATACGTCAACTTCTACCCTGCGTACATTGCGAACCTTAGTGATGACGACCCTATAGACGTAGCTGTGAGTACCAACCGAATAGCAATCCTTAAGGACGCCGTTCCGTTCTCAGAAGAGTTACTCATCTGGTCCGATGAAGCACAATTCGTCCTGAATGCCTCGGGTACTCTCACATCTAAGTCGGTTGAGTTGAACCTAACGACCCAGTTTGACGTAc                                                                                                                                                                                                                                                                                <  1:100018/301‑1 (MQ=255)`  
                              `aaGTATTAACGATGTGTTCTTCTTCCGTAACCGCTTAGGATTCCTTAGTGGGGAGAACATCATATTGAGTCGTACAGCCAAATACTTCAACTTCTACCCTGCGTCCATTGCGAACCTTAGTGATGACGACCCTATAGACGTAGCTGTGAGTACCAACCGAATAGCAATCCTTAAGTACGCCGTTCCGTTCTCAGAAGAGTTACTCATCTGGTCCGATGAAGCACAATTCGTCCTGACTGCCTCGGGTACTCTCACATCTAAGTCGGTTGGGTTGAACCTAACGACCCAGTTTGACATACAg                                                                                                                                                                                                                                                                              <  1:272296/301‑1 (MQ=255)`  
                                `gTATTAACGTTGTGTTCTTCTTCCGTAACCGCTTAGGATTCCTTAGTGGGGAGAACATCATATTGAGTCGTACAGCCAAATACTTCAACTTCTACCCTGCGTCCATTGAGTACCTTAGTGATGACGACCCTATAGACGTAGCTGTTAGTACCAACCGAATAGCAATCCTTAAGTACGCCGTTCCGTTTTCAGAAGAGTTACTCATCTGGCCCGATGATGCTCAATTTGTCCTGACTCTATCGGGTACTCCCACATCTAAGTCGGTTGAGTTGATCCTAACGAACCAGTTTGACGTACTGGa                                                                                                                                                                                                                                                                            >  1:372417/1‑301 (MQ=255)`  
                                `gTATTAACGATGTGTTCTTCTTCCGTAACCGCTTAGGATTCCTTAGTGGGGAGAACATCATATTGAGTCGTACAGCCAAATACTTCAACTTCTACCCTGCGTCCATTGCGAACCTTAGTGATGACGACCCTATAGACGTAGCTGTGATTACCAACCGAATAGCAATCCTTAAGTACGCCGTTCCGTTCTCAGAAGAGTTACTCATCTTGTTCGATTAAGCACAATTCGTCCTGACTACCTCGGTTACTCTCACATCTAAGTCGTTTGGGTTCAACCTAACGACCCCATTTGACGTACATGa                                                                                                                                                                                                                                                                            >  1:452365/1‑301 (MQ=255)`  
                                `gTATTAACGATGTGTTCTTCTTCCGTAACCGCTTAGGATTCCTTAGTGGGGAGAACATCATATTGAGTCGTACAGCCAAATACTTCAACTTCTACCCTGCGTCCATTGCGAACCTTAGTGATGACGACCCTATAGACGTAGCTGTGAGTACCAACCGAATAGCAATCCTTAAGTACGCCGTTCCGTTCTCAGAAGAGTTACTCATCTGGTCCGATGAAGCACAATTCGTCCTGACTGCCTCGGGTACTCTCACATCTAAGTCGGTTGGGTTGAACCTAACGACCCAGTTTGACGTACAGGa                                                                                                                                                                                                                                                                            >  1:86221/1‑301 (MQ=255)`  
                                 `gagacaggATGTGTTCTTCTTCCGTAACCGCTTAGGATTCCTTAGTGGGGAGAACATCATATTGAGTCGTACAGCCAAATACTTCAACTTCTACCCTGCGTCCATTGCGAACCTTAGTGATGACGACCATATAGACGTAGCTGTGAGTACCAACCGAATAGCAATCCTTAAGTACGCCGTTCCGTTCTCAGAAGAGTTACTCATCTGGTCCGATGAAGCACAATTCGTCCTGACTGCCTCGGGTACTCTCACATCTAAGTCGGTTGAGTTGAACCTAACGACCAAGTTTGACGTACAGGAc                                                                                                                                                                                                                                                                           <  1:460079/294‑1 (MQ=255)`  
                                 `agacagcGATGTGTTCTTCTTCCGTAACCGCTTAGGATTCCTTAGTGGGGAGAACATCATATTGAGTCGTACAGCCAAATACTTCAACTTCTACCCTGCGTCCATTGCGAACCTTAGTGATGACGACCCTATAGACGTAGCTGTGAGTACCAACCGAATAGCAATCCTTAAGTACGCCGTTCCGTTCTCAGAAGAGTTACTCATCTGGTCCGATGAAGCACAATTCGTCCTGACTGCCTCGGGTACTCTCACATCTAAGTCGGTTGGGTTGAACCTAACGACCCAGTTTGACGTACAGGAc                                                                                                                                                                                                                                                                           <  1:441012/295‑1 (MQ=255)`  
                                       `gactcggagatgtgtataagaaacagtAGGATTCCTTAGTGGGGAGAACATCATATTGAGTCGTACAGCCAAATACTTCAACTTCTACCCTGCGTCCATTGCGAACCTTAGTGATGACGACCCTATAGACGTAGCTGTGAGTACCAACCGAATAGCAATCCTAAAGTACGCCGTTCCGTTCTCAGAAGAGTTACTCATCTGGTCCGATGAAGCACAATTCGTCCTGACTGCCTCGGGTACTCTCACATCTAAGTCGGTTGGGTTGAACCTAACGACCCAGTTTGACGTACAGGACcgagcg                                                                                                                                                                                                                                                                     <  1:508412/275‑1 (MQ=255)`  
                                                `cttcttcCGTAACCGCTTAGGATTCCTTAGTGGGGAGAACATCATATTGAGTCGTACAGCCCAATACTTCAACTTCTACCCTGCGTCCATTGCGAACCTTAGTGATGACGACCCCATAGACGTATCTGTGAGTACCAACCGAATAGCAATCCTCAAGTACGCCGTTCCGTTCTCCGAAGAGTTACTCATCTTGTCCGATCAAGCTTATTTCGTCCTGCCTGACTCGGTCACTTTCACATCTAATTCGGTTGAGTTAAACCTCACGACACAGTTTGTATTACAGCACCGCCCGTGTCCtttt                                                                                                                                                                                                                                                            >  1:340522/1‑301 (MQ=255)`  
                                                   `cttcCGTAACCGCTTAGGATTCCTTAGTGGGGAGAACATCATATTGAGTCGTACAGACAAATACTTCAACTTCTACCCTGCGTCCATTGCGAACCTTAGTGATGACGACCCTATAGACGTAGCTGTGAGTACCAACCGAATAGCAATCCTTAAGTACGCCGTTCCGTTCTCAGAAGAGTTACTCATCTGGTCCGATGAAGCACAATTGGACCTGACTGCCTCGGTTACTCTCACATCTAAGTAGGTTGGGTTGAACCTAACGACCCAGTTTGACGTACAGGACCGAGCCAGACCTTTTggg                                                                                                                                                                                                                                                         >  1:265167/1‑301 (MQ=255)`  
                                                   `ctgcagTAACCGCTGAGGATGTCTTAGTGGGGAGAACATCATATTGAGTCGTAAAGCCAAATACTTCAACTTCTACCCTGCGTCCATTGCGAACCGTAGTGATGACAACCCTATAGACGTAGCTGTGAGTACCAACCGAATAGCAATCCTTAAGTACGCAGTTTCGTTCTCAGAAGAGTTACTCATCTGGTCCGATGAAGCACAATTCGTCCTGACTGCCTCGGGTACTCTCACATCTAAGTCGGTTGAGTTGAACCTAACGACCCAGTTTGACGTACAGGACCGAGCGAGACCTTTTggg                                                                                                                                                                                                                                                         <  1:229148/296‑1 (MQ=255)`  
                                                    `aagaagtgtataagagacagcgtaaGTGAGGAGAACATCATATTGAGTCGTACAGCCAAATACTTCAACTTCTACCCTGCGTCCATTGCGAACCTTAGAGATGACGACCCGATAGACGTAGCTGTGAGTACCAACCGAATAGCAATCCTTAAGTACGCCGTTCCGTTCTCAGAAGAGTAACTCATCTTGTCCGATGAAGCACAATTCGTCCTGACTGCCTCGGGTACTCTCACATCTAAGTCGGTTGGGTTGAACCTAACGACCCATTTTGACGTACAGGACCGAGCGAGACCTTTTGGGa                                                                                                                                                                                                                                                        <  1:56485/277‑1 (MQ=255)`  
                                                      `ccGTAACCGCTTAGGATTCCTTAGTGGGGAGAACATCATATTGAGTCGTACAGCCAAATACTTCAACTTCTACCCTGCGTCCATTGCGAACCTTAGTGATGACGACCCTATAGACGTAGCTGTGAGTACCAACCGAATAGCAATCCTTAAGTACGCCGTTCCGTTCTCAGAAGAGTTACTAATCTGGTCCGATGAAGCACAATTCGGCCTGACTGCCTCGGGTACTCCCACATCTAAGTCGGTTGAGTTGAACCTAACGACACAGTTTGACGTACAGGACCGAGCGAGACCTTTTgggctg                                                                                                                                                                                                                                                      >  1:120805/1‑298 (MQ=255)`  
                                                           `aCCGCTTAGGATTCCTTAGTGGGGAGAACATCATATTGAGTCGTACAGCCAAATACTTCAACTTCTACCCTGCGTCCATTGCGAACCTTAGTGATGACGACCCTATAGACGTAGCTGTGAGTACCAACCGAATAGCACTCCTTAAGTACGCCGTTCCGTTCTCAGAAGAGTTACTCATCTGGTCCGATGAAGCACAATTCGTCCTGTCTGCCTCGGGTACTCTCACATCTAAGTCGGTTGAGTTGAACCTAACGACCCAGTTTGACGTACAGGACcgagcgctgtctcttatacacatctc                                                                                                                                                                                                                                                 >  1:233475/1‑281 (MQ=255)`  
                                                                     `aTTCCTTAGTGGGGAGAACATCATATTGAGTCGTACAGCCAAATCCTTCAACTTCTACCCTGCGTCCATTGCGAACCTTAGTGATGACGACCCTATAGACGTAGCTGTGAGTACCAACCGAATAGCAATCCTTAAGTACGCCGTTCCGTTCTCAGAAGAGTTACTCATCTGGTCCGATGAAGCACAATTCGTCCTGACTGCCTCGGGTACTCTCACATCTAAGTCGGTTGGGTTGAACCTAACGACCCAGTTTGACGTACAGGACCGAGCGAGACCTTTTGGGATTGGGCGTAATGTCTAc                                                                                                                                                                                                                                       <  1:138305/301‑1 (MQ=255)`  
                                                                     `aTTCCTTAGTGGGGAGAACATCATATTGAGTCGTACAGCCAAATACTTCAACTTCTACCCTGCGTCCATTGCGAACCTTAGTGATGACGACCCTATAGACGTAGCTGTGAGTACCAACCGAATAGCAATCCTTAAGTACGCCGTTCCGTTCTCAGAAGAGTTACTCATCTGGTCCGCTGAAGCACAATTCGTCCTGACTGCCTCGGGTCCTCTCACATCTAAGTCGGTTGGGTTGAACCCAACAACCCAGTTTGACGTACAGGCCCGAGCGAGACCTTTTGGGTTTTGGCGTAATTTCTAc                                                                                                                                                                                                                                       >  1:403322/1‑301 (MQ=255)`  
                                                                          `ttAGTTGGGAGAACATCATATTGAGTCGTACAGCCAAATACTTCAACTTCTACCCTGCGTCCATTGCGAACCTTAGTGATGACGACCCTATAGACGTAGCTGTGAGTACCAACCGAATAGCAATCCTTAAGTACGCCGTTCCGTTCTCAGAAGAGTTACTCATCTGGTCCGATGAAGCACAATTCGTCCTGACTGCCTCGGGTACTCTCACATCTAAGTCGGTTGAGTTGAACCTAACGACCCAGTTTGACGTACAGGACCGAGCGAGACCTTTTGGGATTGGGCGTAATGTCTACTTTGc                                                                                                                                                                                                                                  >  1:201504/1‑301 (MQ=255)`  
                                                                             `gTGGGAAGAACATCGTATTGAGTCGAACAGCCAAATACTTCAACTTCGACACTCCGTCCATTGCGAACCTTAGTGAGGACGACCCTATAGACGGAGCTGTGAGTACCAATCGAATAGCAATACTTAAGTACGCTGTTCCGTGCTCAGAAGAGTTACTCATCTGGTCCGATTAAGCACAATTCGTCCTGACTGCCTCGGGTACTCTCACATCTAAGTCGGTTGGGTTGAACCTAACGACCCAGTTTGACGTACAGGACCGAGCGAGACCTTTTGGGATTGGGCGTAATGTCTACTGTGCTAg                                                                                                                                                                                                                               <  1:309768/301‑1 (MQ=255)`  
                                                                               `ggggAGAACATCATATTGAGTCGTACAGCCAAATACTTCGACTTCTACCCTGCTTCCATTGCGAACCTTAGTGATGACGACCCTATAGACGTAGCTGTGAGTACCAACCGAATAGCAATCCTTAAGTACGCCGTTCCGTTCTCAGAAGAGTTACTCATCTGGTCCGATGAAGCACAATTCGTCCTGACTGCCTCGGGTACTCTCACATCTAAGTCGGTTGGGTTGAACCTAACGACCCAGTTTGACGTACAGGACCGAGCGAGACCTTTTGGGATTGGGCGTAATGTCTACTTTGcctgtc                                                                                                                                                                                                                             <  1:353655/301‑6 (MQ=255)`  
                                                                                    `gaACATCATATTGAGTCGTACAGCCAAATACTTCAACTTCTACCCTGCGTCCATTGCGAACCTTAGTGATGACGACCCTATAGACGTAGCTGTGAGTACCAACCGAATAGCAATCCTTAAGTACGCCGTTCCGTTCTCAGAAGAGTTACTCATCTGGTCCGATGAAGCACAATTCGTCCTGACTGCCTCGGGTACTCTCACATCTAAGTCGGTTGGGTTGAACCTAACGACCCAGTTTGACGTACAGGACCGAGCGAGACCTTTTGGCATTGGGCGTAATGTCTAATTTTCTAGTCCGAgg                                                                                                                                                                                                                        >  1:334652/1‑301 (MQ=255)`  
                                                                                      `aCATCATATTGAGTCGTACAGCCAAATACTTCAACTTCTACCCTGCGTCCATTGCGAACCTTAGTGATGACGACCCTATAGACGTAGCTGTGAGTACCAACCGAATAGCAATCCTTAAGTACGCCGTTCCGTTCTCAGAAGAGTTAATCATCTGGTCCGATGAAGCACAATTCGTCCTGACTGCCTCGGGTACTCTCACATCTAAGTCGGTTGAGTTTAACCTAACGCCCAAGTTTGACGTACAGGACCGAGCGAGACCTTTTGGGATTGGCCGTAATGTCTTCTTTGCTAGTCCGAGGTc                                                                                                                                                                                                                      >  1:469584/1‑301 (MQ=255)`  
                                                                                        `atcatATTGAGTCGTACAGCCAAATACTTCAACTTCTACCCTGCGTCCATTGCGAACCTTAGTGATGACGACCCTATAGACGTAGCTGTGAGTACCAACCGAATCGCAATCATTAAGTACGCCGTTCCGTTCTCAGAAGAGTTACTCATCTGGTCCGATGAAGCACAATTCTTCCTGACTGCCTCGAGTACTCTCACATCTAAGTCGGTTGAGTTGAACCTAACGACCCAGTTTGACGTACAGGACCGAGCGAGACCTTTTGGGATTGGGCGTAATGTATACTTTGCTAGTCAGAGGTCCa                                                                                                                                                                                                                    >  1:387124/1‑301 (MQ=255)`  
                                                                                          `catATTGAGTCGTACAGCCAAATACTTCAACTTCTACCCTGCGTCCATTGAGAACCTTAGTGATGACGACCCTATAGACGTAGCTGTCAGTACCAACCGAATAGCAATCATTAAGTACGCCGTTCCGTTCTCAGAAGAGTTACTCATCTGGTCCGATGAAGCACAATTAGTCCTGACTGCCTCGGGTACTCTCACATCTAAGTCGGTTGAGTTTAACCTAACGACCCAGTTTGACGTACATGACCGAGCGAGACCTTTTGGGATTGGGCGTCATGGCTACTTTGcctatctctgatacaca                                                                                                                                                                                                                  >  1:157820/1‑285 (MQ=255)`  
                                                                                           `atatTGAGTCGTACAGCCAAATACTTCAACTTCTACCCTGCGTCCATTGCGAACCTTAGTGATGACGACCCTATAGACGTAGCTGTGAGTACCAACCGAATAGCAATCCTTAAGTACGCCGTTCCGTTCTCAGAAGAGTTACTCATCTGGTCCGATGAAGCACAATTCGTCCTGACTGCCTCGGGTACTCTCACATCTAAGTCGGTTGGGTTGAACCTAACGCCCAAGTTTGACGTACAGGACCGAGCGAGACCTTTTGGGATTGGGCGGAATTTCAACTTTGCTAGTccctgtctcttat                                                                                                                                                                                                                 >  1:165543/1‑290 (MQ=255)`  
                                                                                           `atatTGAGTCGTACAGCCAAATACTTCAACTTCTACCCTGCGTCCATTGCGAACCTTAGTGATGACGACCCTATAGACGTAGCTGTGAGTACCAACCGAATAGCAATCCTTAAGTACGCCGTTCCGTTCTCAGAAGAGTTACTCATCTGGTCCGATGAAGCACAATTCGTCCTGACTGCCTCGGGTACTCTCACATCTAAGTCGGTTGGGTTGAACCTAACGACCCAGTTTGACGTACAGGACCGAGCGAGACCTTTTGGGATTGGGCGTAATGTCTACTTTGCTAGTCCGACGTCCAGCt                                                                                                                                                                                                                 >  1:423581/1‑301 (MQ=255)`  
                                                                                                          `gCCAAATACTTCAACTTCTACCCTGCGTCCATTGCGAACCTTAGTGATGACGACCCTATAGACGTAGCTGTGAGTACCAACCGAATAGCAATCCTTAAGTACGCCGTTCCGTTCTCAGAAGAGTTACTCATCTGGTCCGATGAAGCACAATTCGTCCTGACTGCCTCGGTGACTCTCACATCTAAGTCGGTTGGGTTGAACCTAACGACCCAGTTTGACGTACAGGACCGAGCGAGACCTTTTGGGATTGGGCGGAATGTATAATTTGCTAGTCCGAGGTCCAGCTTCACGtccatccaac                                                                                                                                                                                                  >  1:240280/1‑299 (MQ=255)`  
                                                                                                          `gCCAAATACTTCAACTTCTACCCTGCGTCCATTGCGAACCTTAGTGATGACGACCCTATAGACGTAGCTGTGAGTACCAACCGAATAGCAATCCTTAAGTACGCCGTTCAGTTCTCAGAAGAGTTACTCATCTGGTCCGATGAAGCACACTTCGTCATGACTGCCTCGGGTACTCTCACATCTAAGTCGGTTGGGTTGAACCTAACGACCCAGTTTGACGTACAGGACCGAGCGAGAACTTTTGGGATTGGGCGTCATGTCTACTTTGCTTGTCCGAGGTCCCTCTTCACTTCCATAcaca                                                                                                                                                                                                  >  1:50028/1‑301 (MQ=255)`  
                                                                                                           `tataagcgacagaCTTCTACCCTGCGTCCATTGCGAACCTTAGTGATGACGACCCTATAGACGTAGCTGTGAGTACCAACCGAATAGCAATCCTTAAGTACGCCGTTCCGTTCTCAGAAGAGTTACTCATCTGGTCCGATGAAGCACAATTCGTCCTGACTGCCTCGGGTACTCTCACATCTAAGTCGGTTGAGTTGAACCTAACGACCCAGTTTGACGTACAGGACCGAGCGAGACCTTTTGGGATTGGGCGTAATGTCTACTTTGCTAGTCCGAGGTCAAGCTTCACGTCCATCCACAg                                                                                                                                                                                                 <  1:436404/289‑1 (MQ=255)`  
                                                                                                               `aTACTTCAACTTCTACCCCGCGTCCTTTGCGAACCTTAGTTATGACGACCCTATAGCCGTAGCTGGGAGTACCAACCGAATAGCAATCCTTAAGTACTCCGTTCCGTGCTCAGAAGAGTTACTCATCTGGTCCGATGAAGCACAATTCGTCCTGACTGCCTCGGGTACTCTCACATCTAAGTCGGTTGGGTTGAACCTAACTACCCAGTTTGACGTACAGGACCGAGCGAGACCTTTTGGGATTGGGCGTAATGTCTACTTTGCTAGTCCGAGGTCCAGCTTCACGTCCATCCACAGGtac                                                                                                                                                                                             <  1:41446/301‑1 (MQ=255)`  
                                                                                                                    `tCAACTTCTACCCTGCGTCCATTGCGAACCTTAGTGATGACGACCCTATAGACGTAGCTGTGAGTACCAACCGAATAGCAATCATTAAGTACGCCGTTCCGTTCTCCGAAGAGTTACTAATCTGGTCCGATGAAGCACAATTCGTCCTGACTGCCTCGGGTACTCTCACATCTAAGTCGGTTGGGTTGAACCTAACGACCCAGTTTGACGTACAGGACCGAGCGAGACCTTTTGGGATTGGGCGTAATGTCTACTTTGCTAGTCCGAGGTCCAGCTTCACGTACATCCACAGGTACTACGc                                                                                                                                                                                        <  1:474336/301‑1 (MQ=255)`  
                                                                                                                    `tCAACTTCTACCCTGCGTCCATTGCGAACCTTAGTGATGACGACCCTATAGACGTAGCTGTGAGTACCAACCGAAGAGCAATCCTTAAGTACGCCGTTCCGTTCTCAGAAGAGTTACTCATCTGGTCCGATGAAGCACAATTCGTCCTGACTGCCTCGGGTACTCTCACATCTAAGTCGGTTGAGTTGAACCTAACGACCCAGTTTGACGTACAGGACCGAGCGAGACCTTTTGGGATTGGGCGTAATGTCTACTTTGCTAGTCCGAGGTCCAGCTTCACGTCCATCCACAGGTACTACGc                                                                                                                                                                                        <  1:24960/301‑1 (MQ=255)`  
                                                                                                                    `agaaCTTCTACCCTGCGTCCATTGCGAACCTTAGTGATGACGACCCTATAGACGTAGCTGTGAGTACCAACCGAATAGCAATCCTTAAGTACGCCGCTCCGTTCTCAGAAGAGTTACTCATCTGGTCCGATGAAGCACAATTCGTCCTGACTGCCTCGGGTACTCTCACATCTAAGTCGGTTTGGTTGAACCTAACGACCCAGTTTGACGTACAGGACCGAGCGAGACCTTTTGGGATTGGGCGTAATGTCTACTTTTCTAGTCCGAGGTCCAGCTTCACGTCCATCCACAGGTACTACGc                                                                                                                                                                                        <  1:180384/299‑1 (MQ=255)`  
                                                                                                                    `acagctgcTACCCTGCGGCCATTGCGAACCTTAGTGATGACGACCCTATAGACGTAGCTGTGAGTACCAACCGAATAGCAATCCTTAAGTACGCCGTTCCGTTCTCAGAAGAGTTACTCATCTGGTCCGATGAAGCACAATTCGTCCTGACTGCCTCGGGTACTCTCACATCTAAGTCGGTTGGGTTGAACCTAACGACCCAGTTTGACGTACAGGGCCGAGCGAGACCTTTTGGGATTGGGCGTAATGTCTACTTTGCTAGTCCGAGGTCCAGCTTCACGTCCATCCACAGGTACTACGc                                                                                                                                                                                        <  1:107938/294‑1 (MQ=255)`  
                                                                                                                    `acagcTTCTACCCTGCGTCCATTCCGAAACTTAGTGATGACGACCCTATAGACGTAGCTGCGAGTACCAAACAAATAGCAATCATTAAGGACGCCGTTACGTTCTCAGAAGAGGTACTCATCTGGTCCGATGAAGCACAATTCGTCCTGACTGCCTCGGGTACTCTCACAGCTAAGTCGGTTGGGTTGAACCTAAAGACCCAGTTTGACGTACAGGAACGAGCGAGACCTGTTGGGATTGGGCGTAATGTCTACTTTGCTAGTCCGAGGTCCAGCTTCACGTCCATCCACAGGTACTACGc                                                                                                                                                                                        <  1:34965/297‑1 (MQ=255)`  
                                                                                                                        `cTTCTACCCTGCGTCCATTGCGAACCTTAGTGATGACGACCCTATATACATATCTGTGAGTACCAACCGAATAGCAATCCTTAAGTACGCCGTTCCGTTCTCAGAAGATTTACTCATCTGGTCCGATGAAGTACAATTCGTCCTGACTGTCTCGGGTACTCTCCCATCTAAGTCGGTTGGATTGAACCTAACGACCCAGTTTGACGTACCGGACCGAGCGAGACCTTTTGGGATTATGCGTAATGTCTACTTTGCTAGTCCGAGGTCCAGCTTCACGtccatcccccggaacctgcctctt                                                                                                                                                                                    >  1:211684/1‑284 (MQ=255)`  
                                                                                                                        `cTTCTACCCTGCGTCCATTGCGAACCTTAGTGATGACGACCCTATAGACGTAGCTGTGAGTACCAACCGAATAGCAATCCTTAAGTACGCCGTTCCGTTCTCAGAAGAGTTACTCATCTGGACCGATGAAGCACAATTCGTCCTGACTGCCTCGGGTACTCTCACATCTAAGTCGGTTGGGTTGAACCTAACGACCCAGTTTGACGTACAGGACCGAGCGAGACCTTTTGGGATTGGGCGTAATGTCTACTTTGCTAGTCCGAGGTCCAGCTTCACGTCCATCCACAGGTACTACGCtgtg                                                                                                                                                                                    >  1:168822/1‑301 (MQ=255)`  
                                                                                                                            `tACCCTGCGTCCATTGCGAACCTTAGTGATGACGACCCTATAGACGTAGCTGTTAGTACCAACCGAATAGCAATCCTTAAGTACGCCGTTCCGTTCTCAGAAGAGTTACTCATCTGGTCCGATGAAGCACAATTCGTCCTGACTGCCTCGGGTACTCTCACATCTAAGTCGGTTGGGTTGAACCTAACGACCCAGTTTGACGTACAGGACCGAGCGAGACCTTTTGGGATTGGGCGTAATGTCTACTTTGCTAGTCCGAGGTCCAGCTTCACGTCCATCCACAGGTACTACGCTGTGCAgg                                                                                                                                                                                <  1:358845/301‑1 (MQ=255)`  
                                                                                                                            `tACCCTGCGTCCATTGCGAACCTTAGTGATGACGACCCTATAGACGTAGCTGTGAGTACCAACCGAATAGCAATCCTTAAGTACGCCGTTCCGTTATCAGAAGAGTTACTCATCTGGTCCGATGAAGCACAATTCGTCCTGACTGCCTCGGGTACTCTCACATCTAAGTCGATTGAGTTGAACCTAACGACCCAGTTTGACGTACAGGACCGAGCGAGACCTTTTGGGATTGGGCGTAATGTCTACTTTGCTAGTCCGAGGTCCAGCTTCACGTCCATCCACAGGTACTACGCTGTGCAgg                                                                                                                                                                                <  1:221923/301‑1 (MQ=255)`  
                                                                                                                              `cccTGCGTCCATTGCGAACCTTAGTGATGACGACCATATAGACGTAGCTGTGAGTACCACCCGAATAGCAATCCTTAAGTACGCCGTTCCGTGCTCAGCAGAGTTACTAATCTGGTCCGATGAAGCACAATTCGTCCTGACTGCCTCGGGTACTCTCACATCTAAGTCGGTTGAGTTGAACCTAACGACCCAGTTTGACGTACAGGACCGAGCGAGACCTTTTGGGATTGGGCGTAATGTCTACTTTGCTAGTCCGAGGTCCAGCTTCACGTCCATCCACAGGTACTACGCTGTGCAGGAt                                                                                                                                                                              <  1:141810/301‑1 (MQ=255)`  
                                                                                                                                 `ggCGTCCATGGCCAACCTTAGTGATAACGACCCGCTATACGTAGCTGTGCGTACCAACCGAATAGCAATCCTTAAGTACGCCGTTCCGTTCTCAGAAGAGTTACTCATCTGGTCCGATGAAGCACAACTCGTCCTGACTGCCTCGGGGTCTCTCACATCCAAGTCGGTTGAGTTGAACCTAACGACCTAGTTTGGCGTACAGGACCGAGCGAGACCTTTTGGGATTGGGCGTAATGTCTACTTTGCTAGTCCGAGGTCCAGCTTCACGTCCATCCACAGGTACTACGCTGTGCAGGATGTc                                                                                                                                                                           <  1:83229/300‑1 (MQ=255)`  
                                                                                                                                      `ccATTGCGAAACTTCGTGATGACGACCCTATAGACGTAGCTGTGAGTACCAACCGAATAGCAATCCTTAAGTACGCCGTTCCGTTCTCAGAAGAGTTACTCATCTGGTCCGATGAAGCACAATTCGTCCTGACTGCCTCGGGTACTCTCACATCTAAGTCGGTTGGGTTGAACCTAACGACCCAGTGTGACGTACAGGATCGAGCGAGACCTGTTGGGATTGGGCGTAATGTCTACTTTGCTAGTCCGAGGTCCAGCTTCACGTCCATCCACAGGTACTACGCTGTGCAGGATGTCAGTTc                                                                                                                                                                      <  1:70021/301‑1 (MQ=255)`  
                                                                                                                                        `aTTGCGAACCTTAGTGATGACGACCCTATAGACGTAGCTGTGAGTACCAACCGAATAGCAATCCTTAAGTACGCCGTTCCGTTCTCAGAAGAGTTACTCATCTGGTCCGATGAAGCACAATTCGTCCTGACTGCCTCGGGTACTCTCACATCTAAGTCGGTTGGGTTGAACCTAACGACCCAGTTTGACGTACAGGACCGAGCGAGACCTTTTGGGATTGGGCGTAATGTCTACTTTGCTAGTCCGAGGTCCAGCTTCACGTCCATCCACCGGTACTACGCTGTGCAGGATGTCAGTTCCg                                                                                                                                                                    >  1:225506/1‑301 (MQ=255)`  
                                                                                                                                                `ccTTAGTGATGACGACCCTATAGACGTAGCTGTGAGTACCAACCGAATAGCAATCCTTAAGTACGCCGTTCCGTTCTCAGAAGAGTTACTCATCTGGTCCGATGAAGCACAATTCGTCCTTACTGCCTCGGGTACTCTCACATCTAAGTCGGTTGGGTTGAACCTAACTACCCAGTTTGACGTACATGACCGAGCGAGACCTTTTGGGATTGGGCGTAATGTCTACTTTGCTAGTCCGAGGTCCAGCTTCACGTCCATCCACAGGTACTACGCTGTGCAGGAtctgtctcttatacacatc                                                                                                                                                            >  1:11714/1‑283 (MQ=255)`  
                                                                                                                                                `ccTTAGTGATGACGACCCTATAGACGTAGCTGTGAGTACCAACCGAATAGCAATCCTTAAGTACGCCGTTCCGTTCTCAGAAGAGTTACTCATCTGGTCCGATGAAGCACAATTCGTCCTGACTGCCTCGGGTACTCTCACATCTAAGTCGGTTGGGTTGAACCTAACGACCCAGTTTGACGTACAGGACCGAGCGAGACCTTTTTGGATTGGGCGTAATGTCTACTTTGCTAGTCCGAGGTCCAGCTTCATGTCCATCCACAGGTACTACGCTGTTCAGGATGTCCGTTCCGTTAAGAAt                                                                                                                                                            >  1:214750/1‑301 (MQ=255)`  
                                                                                                                                                `ccTTAGTGATGACGACCCTATAGACGTAGCTGTGAGTACCAACCGAATAGAAATCCTTAAGTACGCCGTTCAGTTCTCAGAAGAGTTACTCATCTAGTCAGATGAAGCACAATTTATCCTGACTGCCTCGGGTACTATCATATCTAAGTCGGTTGAGTTGAACCTAACGACACAGTTTGACTTAAAGGCCCGAGCGAGACCTATTGAGATTGAGCGTAATGTCTACTTTGCTACTCCGATGTAAAAATTCACGTCCATAAACAGGTACTAAGCGGTGGAGGATGTAAgctgtctcttatac                                                                                                                                                            >  1:75320/1‑288 (MQ=255)`  
                                                                                                                                                            `aaaccggattgACGTAGCTGTGAGTACCAACCGAATCGCAATCCTTCATTACTCCGTTCCGTTCTCATAAGAGTTATTCTTCTGGTTCAATGATGCACAATTCGTCCTGACTGCCGCGGGTACTCTCACATCTAAGGCGGTTGGTTTTACCCTAACGACCCAGTTTGACGTACAGGACCGAGAGAGACCAGTTGGGATTGGGCGTAATTTCTACTTTGCTAGTCCGAGGTCCAGCTTCACGTCCATCCACAGGTACTACGCTGTGCAGTATGTCAGTTCCGTTAAGAATGCTGAGGACAtt                                                                                                                                                <  1:432993/291‑1 (MQ=255)`  
                                                                                                                                                              `acCCTATAGACGTAGCTGTGAGTACCAACCGAATAGCAATCCTTAAGTACGCCGTTCCGTTCTCAGAAGAGTTACTCATCTGGTCCGATGAAGCACAATTCGTCCTGACTGCCTCGGGTACTCTCACATCTAAGTCGGTTGGGTTGAACCTAACGACCCAGTTTGACGTACAGGACCGAGCGAGACCTTTTGGGATTGGGCGTAATGTCTACTTTGCTAGTCCGAGGTCCAGCTTCACGTCCATCCACAGGTACTACGCTGTGCAGGATGTCAGTTCCGTTAAGAATGCTGAGGACATTAc                                                                                                                                              >  1:177566/1‑301 (MQ=255)`  
                                                                                                                                                                `agagacagcGTAGCTGTGAGTACCAACCGAATAGCAATCCTTAAGTACGCCGTTCCGTTCTCAGAAGAGTTACTCATCTGGTCCGATGAAGCACAATTCGTCCTGACTGCCTCGGGTACTCTCACATCTAAGTCGGTTGGGTTGAACCTAACGACCCAGTTTGACGTACAGGACCGAGCGAGACCTTTTGGGATTGGGCGTAATGTCTACTTTGCTAGTCCGAGGTCCAGCTTCACGTCCATCCACAGGTACTACGCTGTGCAGGATGTCAGTTCCGTTAAGAATGCTGAGGACATTACAt                                                                                                                                            <  1:134366/293‑1 (MQ=255)`  
                                                                                                                                                                 `cTATAGACGTAGCTGTGAGTACCAACCGAATAGCAATCCTTAAGTACTCCGTTCCGTTCTCAGAAGAGTTACTCATCTGGTCCGATGAAGCACAATTCGTCCTGACTGCCTCGGGTACTCTCACATCTAAGTCGGTTGAGTTGAACCTAACGACCCAGTTTGACGTACAGGACCGAGCGAGACCTTTTGGGATTGGGCGTAATGTCTACTTTGCTAGTCCGAGGTCCAGCTTCACGTCCATCCACAGGTACTACGCTGTGCAGGATGTCAGTTCCGTTAAGAATGCTGAGGACATTACATc                                                                                                                                           >  1:172739/1‑301 (MQ=255)`  
                                                                                                                                                                 `cTATAGACGTAGCTGTGAGTACCAACCGAATAGCAATCCTTAAGTACGCCGTTCCGTTCTCAGAAGAGTTACTCATCTGGTCCGATGAATCACAATTCGTCCTGACTGCCTAGGGTACTCTCACATCTAAGTCGGTTGGGTTGAACCTAACGACCCAGTTTGACGTACAGGACGGAACGAGACCTTTTAGGATTGGGCGTAATGTCTACTTTGCTAGTCCGAGGGCCAGCTTCACGTCCATCCACAGGTACTACGCTGTGCAGGATGTCAGTTCCGTTAAGAATGCTGAGGCCATTACATc                                                                                                                                           >  1:363799/1‑301 (MQ=255)`  
                                                                                                                                                                 `cTATAGACGTAGCTGTGAGTACCAACCGAATAGCAATCCTTAAGTACGCCGTTCCGTTCTCAGAAGAGTTACTCATCTGGTCCGATGAAGCACAATTCGTCCTGACTGCCTCGGGTACTCTCACATCTAAGTCGGTTGGGTTGAACCTAACGACCCAGTTTGACGTACAGGACCGAGCGAGACCTTTTGGGATTGGGCGTAATGTCTACTTTGCTAGTCCGAGGTCCAGCTTCACGTCCATCCACAGGTACTACGCTGTGCAGGATGTCAGTTCCGTTAAGAATGCTGAGGACATTACATc                                                                                                                                           >  1:397620/1‑301 (MQ=255)`  
                                                                                                                                                                 `cTATAGACGTAGCTGTGAGTACCAACCGAATAGCAATCCTTAAGTACGCCGTTCCGTTCTCAGAAGAGTTACTCATCTGGTCCGATGAAGCACAATTCGTCCTGACTGCCTCGGGTACTCTCACATCTAAGTCGGTTGGGTTGAACCTAACGACCCAGTTTGACGTACAGGACCGAGCGAGACCTTTTGGGATTGGGCGTAATGTCTACTTTGCTAGTCCGAGGTCCAGCTTCACGTCCATCCACAGGTACTACGCTGTGCAGGATGTAAGTTCCGTTAAGAATGCTGAGGACATTACATc                                                                                                                                           >  1:103677/1‑301 (MQ=255)`  
                                                                                                                                                                 `cTATAGACGTAGCTGTGAGTACCAACCGAATAGCAATCCTTAAGTACGCCGTTCCGTTCTCAGAAGAGTTACTCATCTGGTCCGATGAAGCACAATTCGTCCTGACTGCCTAGGGTACTCTCACATCTAAGTCGGTTGGGTTGAACCTAACGACCCAGTTTGACGTACAGGACCGAGCGAGACCTTTTGGGATTGGGCGTAATGTCAACTTTGCTAGTCCGAGGTACAGCTTCACGTCCATCCACAGGTACTACGCTGTGCAGGATGTCAGTTCCGTTAAGAATGCTGAGGACATTACATc                                                                                                                                           <  1:484147/301‑1 (MQ=255)`  
                                                                                                                                                                     `aGACGTAGCTGTGAGTACCAACCGAATAGCAATCCTTAAGTACGCCGTTCCGTTCTCAGAAGAGTTACTCATCTGGTCCGATGAAGCACAATTCGTCCTGACTGCCTCGGGTACTCTCACATCTAAGTCGGTTGGGTTGAACCTAACGACCCAGTTTGACGTACAGGACCGAGCGAGACCTTTTGGGATTGGGCGTAATGTCTACTTTGCTAGTCCGAGGTCCAGCTTCACGTCCATCCACAGGTACTACGCTGTGCAGGATGTCAGTTCCGTTAAGAATGCTGAGGACATTACATCacac                                                                                                                                       <  1:149929/301‑1 (MQ=255)`  
                                                                                                                                                                     `aGACGTAGCTGTGAGTACCAACCGAATAGCAATCCTTAAGTACGCCGTTCCGTTCTCAGAAGAGTTACTCATCTGGTCCGATGAAGCACAATTCGTCCTGACTGCCTCGGGTACTCTCACATCTAAGTCGGTTGGGTTGAACCTAACGACACAGTTTGACGTACAGGACCGAGAGAGACATTTTGGGATTGGGCGTAATGTCTACTTTGCGAGTCAGAGGTCCAGCTTCACGTCCATCCACAGGTACTACGCTGTGCAGGATGTCAGTTCCGTTAAGAATGCTGAGGACATTACATCacac                                                                                                                                       <  1:161806/301‑1 (MQ=255)`  
                                                                                                                                                                      `gACGTAGCTGTGAGTACCAAACGAATAGCAATTCTTAAGTAAGACGTTCCGTTCTCAGAAGAGTTACTCATCTGGTCCGATGAAGCACAATTCGTCCTGACTGCCTCGGGTACTCTCACATCTAAGTCGGTTGGGTTGAACCTAACGACCCAGTTTGACGTACAGGACCGAGCGAGACCTTTTGGGATTGGGCGTAATGTCTACTTTGCTAGTCCGAGGTCCAGCTTCACGTCCATCCACAGGTACTACGCTGTGCAGGATGTCAGTTCCGTTAAGAATGCTGAGGACATTACATCACACg                                                                                                                                      <  1:294751/301‑1 (MQ=255)`  
                                                                                                                                                                          `tAGCTTTGAGTACCAACCGAATAGCAATCCTTAAGTACGCCGTTCCGTTCTCAGAAGAGTTACTCATCTGGTCCGATGGAGCACAATTCGTCCTGACTGCCTCGGGTACTCTCACATCTAAGTCGGTTGAGTTGAACCTGACGACCCAGTTTGACGTACAGGACCGAGCGAGACCTTTTGGGATTGGGCGTAATGTCTACTTTGCTAGTCCGAGGTCCAGCTTCACGTCCATCCACAGGTACTACGCTGTGCAGGATGTCAGTTCCGTTAAGAATGCTGAGGACATTACATCACACGTTcc                                                                                                                                  <  1:275446/301‑1 (MQ=255)`  
                                                                                                                                                                            `gCTGTGAGTACCAACCGAATAGCAATCCTTAAGTACGCCGTTCCGTTCTAAGAAGAGTTACTCATCTGGTCCGATGAAGCACAATTCGTCCTGACTGCCTCGGGTACTCTCACATCTAAGTCGGTTGAGTTGAACCTAACGACCCAGTTTGACTTACAGGACCGAGCGAGACCTTTTGGGATTTGGCGTAATGTCTACTTTGCTAGTCCGAGGTCCAGCTTCACGTCCATCCACAGGTACTACGCTGTGCAGGATGTCAGTTCCGTTAAGAATGCTGCGGAcctgtctcttatacacatct                                                                                                                                >  1:79709/1‑282 (MQ=255)`  
                                                                                                                                                                                          `ccGAATAGCAATCCTTAAGTACGCCGTTCCGTTCTCAGAAGAGTTACTCATCTGGTCCGATGAAGCACAATTCGTCCTGACTGCCTCGGGTACTCTCACATCTAAGTCGGTTGAGTTGAACCTAACGACCCAGTTTGACGTACAGGACCGAGCGAGACCTTTTGGGATTGGGCGTAATGTCTACTTTGCTAGTCCGAGGTCCAGCTTCACGTCCATCCACAGGTACTACGCTGTGCAGGATGTCAGTTCCGTTAAGAATGCTGAGGACATTACATCACCCGTTCCTAACTACATCCCTAAt                                                                                                                  >  1:418344/1‑301 (MQ=255)`  
                                                                                                                                                                                            `tgggctcggagatgtgtataagagacagggtctcAGAAGAGTTACTCATCTGGTCCGATGAAGCACAATTCGTCCTGACTGCCTCGGGTACTCTCACATCTAAGTCGGTTGAGTTGAACCTAACGACCAAGTTTGACGTACAGGACCGAGCGAGACCTTTTGGGATTGGGCGTAATGTCTACTTTGCTAGTCCGAGGTCCAGCTTCACGTCCATCAACAGGTACTACGCTGTGCAGGATGTCAGTTCCGTTAAGAATGCTGAGGACATTACATCACACGTTCCTAACTACATCCCTAATgg                                                                                                                <  1:345939/271‑1 (MQ=255)`  
                                                                                                                                                                                            `gAATAGCAATCCTTAAGTACGCCGTTCCGTTCTCAGAAGAGTTACTCATCTGGTCCGATGAAGCACAATTCGTCCTGACTGCCTCGGGTACTCTCACATCTAAGTCGGTTGGGTTGAACCTAACGACCCAGTTTGACGTACAGGACCGAGCGAGACCTTTTGGGATTGGGCGTAATGTCTACTTTGCTAGTCCGAGGTCCAGCTTCACGTCCATCCACAGGTACTACGCTGTGCAGGATGTCAGTTCCGTTAAGAATGCTGAGGACATTACATCACACGTTCCTAACTACATCCCTAATgg                                                                                                                >  1:392061/1‑301 (MQ=255)`  
                                                                                                                                                                                            `gAATAGCAATCCTGAATTACGCCGTTCCGTTCCCAGAAGAGTTACGCATCTGGTCCGATGAAGCACAATTCGTCCTGACTTCCTCGGGTACTCTCACATCTAGGTCGGTTGAGTTGAACCTAACGGCCCAGTTTGACGTACAGGACCGAGCGAGACCTTTTGGGATTGGGCGTAATGTCTACTTTGCTAGTCCGAGGTCCAGCTTCACGTCCATCCACAGGTACTACGCTGTGCAGGATGTCAGTTCCGTTAAGAATGCCGAGGACATTACATCACACGTTCCTAACTACATCCCTAATgg                                                                                                                <  1:244464/301‑1 (MQ=255)`  
                                                                                                                                                                                            `aaaTAGCAATCCATCAGTACTCCGTTCCGTGCTCAGAAGAGTTACTCATCTGGTCCTATAAAGCAGAATTCGTCCTGTGTGCCTCCGGTACTGGCACATCTAAGTCGGTTGGGTTGAAGCTAACGTCCCAGTTTGCCTAACAGGACCGAGCGAGACCTGTTGGGATTGGACGTAATGTGTACTGAGCTAGTCCGAGGTCCAGCCTAACGTCCATCCACAGGTACTAAGCTGTGCAGGATGTAAGTTCCGTTAAGGATGCTGAGAACATTACATCACACGTTCCTAACTACATCCCTAATgg                                                                                                                <  1:268413/300‑1 (MQ=255)`  
                                                                                                                                                                                                   `aaTCCTTGAGTACGCCGTGCCGTTCTCAGAAGAGTTACTCATCTGGTCCGATGAAGCACAATTCGTCCTGACTGCCTCGGGTACTCTCACATCTAAGTCGGTTGGGTTGAACCTAACGACCCAGTTTGACGTACAGGACCGAGCGAGACCTTTTGGGATTGGGCGTAATGTCTACTTTGCTAGTCCGAGGTCCAGCTTCACGTCCATCCACAGGTACTACGCTGTGCAGGATGTCAGTTCCGTTAAGAATGCTGAGGACATTACATCACACGTTCCTAACTACATCCCTAATGGTGTGTTc                                                                                                         <  1:112892/301‑1 (MQ=255)`  
                                                                                                                                                                                                      `ccTTAAGTACGCCGTTCCGTTCTCAGAAGAGTTACTCATCTGGTCCGATGAAGCACAATTCGTCCTGACTGCCTCGGGTACTCTCACATCTAAGTCGGTTGAGTTGAACCTAACGACCCAGTTTGACGTCCAGGACCTAGCGAGACCTTTTTGGATTGGGCGTAATGTCTACTTTGCTAGTCCGAGGTCCAGCTTCACGTCCATCCACAGGTACTACGCTGTGCATGATGTCAGTTCCGTTACGAATGCTGAGGACATTACATCAAACGCTCCTAACTACATCCCTAATgcctgtctcttt                                                                                                      >  1:288364/1‑290 (MQ=255)`  
                                                                                                                                                                                                       `cTTAAGTACGCCGTTCCGTTCTCAGAAGAGTTACTCATCTGGTCCGATGAAGCACAATTCGTCCTGACTGCCTCGGGTACTCTCACATCTAAGTCGGTTGAGTTGAACCTAACGACCCAGTTTGAAGTACAGGACCGAGCGAGACCTTTTGGGATTGGGCGTAATGTCTACTTTGCTAGTCCGAGGTCCAGCTTCACGTCCATCCACATGTACTACGCTGTGCAGGATGTCAGTTCCGTTAAGAATGCTGAGGACATTACATCACACGTTCCTAACTACATCCCTAATGGTGTGTTCAGTa                                                                                                     <  1:145750/301‑1 (MQ=255)`  
                                                                                                                                                                                                        `ttAAGTACGCCGTTCCGTTCTCAGAAGAGTTACTCATCTGGTCCGATGAAGCACAATTCGTCCTGACTGCCTCGGGTACTCTCACATCTAAGTCGGTTGCGTTAAACCTAACGACCCAGTTTGACGTACAGGACCGAGCGAGACCTTTTGGGATTGGGCGTAATGTCTACTTTGCTAGTCCGAGGTCCAGCTTCACGTCCATCCACAGGTACTACGCTGTGCAGGATGTCAGTTCCGTTAAGAATGCTGAGGACATTACATCACACGTTCCTAACTACATCCCTAATGGTGTGTTCAGTAt                                                                                                    <  1:428143/301‑1 (MQ=255)`  
                                                                                                                                                                                                               `cGCCGTGCCGTTCTCAGAAGAGTTACTCATCTGGTCCGATGAAGCACAATTCGTCCTGACTGCCTCGGGTACTCTCACATCTAAGTCGGTTGAGTTGAACCTAACGACCCAGTTTGACGTACAGGACCGAGCTAGACCTTTTGGGATTGGGCGTAATGTCTACTTTGCTAGTCCGAGGTCCAGCTTCACGTCCATCCACAGGTACTACGCTGTGCAGGATGTCAGTTCCGTTAAGAATGCTGAGGACATTACATCACACGTTCCTAACTACATCCCTAATGGTGTGTTCAGTATTTGCGGa                                                                                             <  1:207107/301‑1 (MQ=255)`  
                                                                                                                                                                                                                            `tcAGAAGAGTTACTCATCTGGTCCGATGAAGCACAATTCGTCCTGACTGCCTCGGGTACTCTCACATCTAAGTCGGTTGAGTTGAACCTAACGACCCAGTTTGACGTACAGGACCGAGCGAGACCTTTTGGGATTTGGCGTAATGTCTACTTTGCTAGTCCGAGGTCCAGCTTCACGTCCATCCACAGGTACTACGCTGTGCAGGATGTCAGTTCCGTTAAGAATGCTGAGGACATTACATCACACGTTCCTAACTACATCCCTAATGGTGTGTTCAGTATCTgtctcttatacacatctc                                                                                >  1:90037/1‑284 (MQ=255)`  
                                                                                                                                                                                                                                  `gagTTACTCATCTGGTCCGATGAAGCACAATTCGTCCTGACTGCCTCGGGTACTCTCACATCTAAGTCGGTTGGGTTGAACCTAACGACCCAGTTTGACGTACAGGACCGAGCGAGACCTTTTGGGATTGGGCGTAATGTCTACTTTGCTAGTCCGAGGTCCAGCTTCACGTCCATCCACAGGTACTACGCTGTGCAGGATGTCAGTTCCGTTAAGAATGCTGAGGACATTACATCACACGTTCCTAACTACATCCCTAATGGTGTGTTCAGTATTTGCGGAAGTGGTACGGAAAACTTCt                                                                          >  1:464813/1‑301 (MQ=255)`  
                                                                                                                                                                                                                                          `cATCTGGTCCGATGAAGCACAATTCGTCCTGACTGCCTCGGGTACTCTCACATCTAAGTCGGTTGGGTTGAACCTAACGACCCAGTTTGACGTACAGGACCGAGCGAGACCTTTTGGGATTGGGCGTAATGTCTACTTTGCTAGTCCGAGGTCCAGCTTCACGTCCATCCACAGGTACTACGCTGTGCAGGATGTCAGTTCCTTTAAGAATGCTGCGGACATTACATCACACGTTCCTAACTACATCCCTAATGGTGTGTTCAGTATTTGCGGAAGTGGTACGGAAAACTTATGTTCGGTa                                                                  >  1:194698/1‑301 (MQ=255)`  
                                                                                                                                                                                                                                           `aTCTGGTCCGATGAAGCACTATTCGTCCTGACTGCCTCGTGTACTCTCACATCTAAGTCGGTTAGGTTGAACCTAACGACCCAGTTTGACGTACAGGACCGAGCGAGACCTTTTGGGATTGGGCGTAATGTCTACTTTGCTAGTCCGAGGTCCATCTTCACGTCCATCCACAGGTACTACGCTGTGCAGGATGTCAGTTCCGTTAAGAATGCTGAGGACATTACATCACACGTTCCTAACTACATCCCTAATGGTGTGTTCAGTATTTTCGGAAGTGGTACGGAAAACTTCTGTTCGGTAc                                                                 <  1:217678/301‑1 (MQ=255)`  
                                                                                                                                                                                                                                              `tGGTCCGATGAAGCACAATTCGTCCTGACTGCCTCGGGTACTCTCACATCTAAGTCGGTTGAGTTGAACCTAACGACCCAGTTTGACGTACAGGACCGAGCGAGACCTTTTGGGATTGGGCGTAATGTCTACTTTGCTAGTCCGAGGTCCAGCTTCACGTCCATCCACAGGTACTACGCTGTGCAGGATGTCAGTTCCGTTAAGAATGCTGAGGACATTACATCACACGTTCCTAACTACATCCCTAATGGTGTGTTCAGTATTTGCGGAAGTGGTACTGAAAACTTCTGTTCGGTACTAt                                                              <  1:382184/301‑1 (MQ=255)`  
                                                                                                                                                                                                                                               `gttCCGCTTAAGCATCATTCGTCCTTACTGCCTCGGGTACTCTCACTTCTAAGTCGGTTGAGTTGAACCTAACGACCCAGTTTTACGTACAGGACCTAGCGATACCTTTTGGGATTGGGCGTAATGTCTACTTTGCTAGTCCGAGGTCCAGCTTCACGTCCATCCACAGGTACTACGCTGTGCAGGATGTCAGTTCCGTTAAGAATGCTGAGGACATTACATCACACGTTCCTAACTACATCCCTAATGGTGTGTTCAGTATTTGCGGAAGTGGTACGGAAAACTTCTGTTCGGTACTAtc                                                             <  1:315255/299‑1 (MQ=255)`  
                                                                                                                                                                                                                                               `ggTCCGATGAAGCACAATTCGTCCTGACTGCCTCGGGTACTCTCACATCTAAGTCGGTTGGGTTGAACCTAACGACCCAGTTTGACGTACAGGACCGAGCGAGACCTTTTGGGATTGGGCGTAATGTCTACTTTGCTAGTCCGAGGTCCAGCTTCACGTCAATCCACAGGTACTACGCTGTGCAGGATGTCAGTTCCGTTAAGAATGCTGAGGACATTACATCACACGTTCATAACTACATCCCTAATGGTGTGTTCAGTATTTGCGGAAGTGGTACGGAAAACTTCTGTTCGGTACTAtc                                                             <  1:410666/301‑1 (MQ=255)`  
                                                                                                                                                                                                                                                 `tCCGTTGAAGCACAATTCGTTCTGACTTCCTCGGGTAAGCTCACATCTAAGTCGGTTGGGTTGAACCTAACGACCCAGTTTGACGTACAGGACCGAGCGAGACCTTTTGGGATTGGGCGTAATGTCTACTTTGCTAGTCCGAGGTCCAGCTTCACGTCCATCCACAGGTACTACGCTGTGCAGGATGTCAGTTCCGTTAAGAATGCTGAGGACATTACATCACACGTTCCTAACTACATCCCTAATGGTGTGTTCAGTATTTGCGGAAGTGGTACGGAAAACTTCTGTTCGGTACTAtctc                                                           <  1:95988/301‑1 (MQ=255)`  
                                                                                                                                                                                                                                                   `cGATGAAGCACAATTCGTCCTGCCTGCCTCTGGTACTCTCACCTCTAAGTCGTTTGAGTTGAACCTAACGACCCAGTTTGACGTACAGGACCGAGCGAGAACTTTTGGGATTGGTCGTAATGTCTACTTTGCTAGTCCGAGGTCCAGCTTCACGTCCATCCACAGGTACTACGCTGTGCAGGATGTCAGTTCCGTTAAGAATGCTGAGGACATTACATCACACGTTCCTAACTACATCCCTACTGGTGTGTTCAGTATTTGCGGAAGTGGTACGGAAAACTTCTGTTCGGTACTATCTCAc                                                         <  1:449300/301‑1 (MQ=255)`  
                                                                                                                                                                                                                                                             `caATTCGTCCTGACTGCCTCGGGTACTCTCACATCTAAGTCGGTTGAGTTGAACCTAACGACCCAGTTTGACGTACAGGACCGAGCGAGACCTTTTTGGATTGGGCGTAATGTCTACTTTGCTAGTCCGAGGTCCAGCTTCACGTCCATCCACAGGTACTACGCTGTGCAGGATGTCAGTTCCGTTAAGAATGCTGAGGACATTACATCACACGTTCCTAACTACATCCCTAATGGTGTGTTCAGTATTTGCGGAAGTGGTACGGAAAACTTCTGTTCGGTAcctgtctcttatacacatt                                               >  1:479368/1‑283 (MQ=255)`  
                                                                                                                                                                                                                                                                 `tCGTCCTGGCTGCCTCGGGTAGTCTGGCAGCAAAATCGCATGGGTTGAACCTAACGACCCAGTTAGTCGTACAGTACCGAGCGAGACCGTTTGGGATTGGGTGTAATATCTACTTTTGTAGTCCGAGATCCAGCTTCACGTCCATCCGCAGGTATTACGCTGTGCAGGATGTCAGTTCCGTTAATAATGCTGAGGACATTACATCGCACGTTCCTAACTACATCCCTAATGGTGTGTTAAGTAGTTGAGGAAGTGGTACGGAAAACTTCTGTTCGGTACTATCTCACGGGGACCCTAGTaa                                           <  1:368617/301‑1 (MQ=255)`  
                                                                                                                                                                                                                                                                 `tCGTCCTGAATGCCTCGGGTACTCTCACATCTAAGTCGGTTGGGTTGAACCTAACGACCCAGTTTGACGTACAGGACCGAGCGAGACCTTTTTGGATTGGGCGTAATGTCTACTTTGCTAGTCCGAGGTCCAGCTTCACGTCCATCCACAGGTACTACGCTGTGCAGGATGACAGTTCCGTTAAGAATGCTGAGGACATTACATCACACGTTCCTAACTACATCCCTAATGGTGTGTTCAGTATTTGCGGAAGTGGTACTGAAAACTTCTGTTCGGTACTATCTCACGGGGACCCTAGTaa                                           <  1:15426/301‑1 (MQ=255)`  
                                                                                                                                                                                                                                                                           `ctgctctGGTTCTCTCACATCTAAGTCGGTTGGGTTGAACCTACCGACCCAGTTTGACGTACAGGACCGAGCGAGACCTTTTGGGATTGGGCGTAATGTCTACTTTGCTAGTCCGAGGTCCAGCTTCACGTCCATCCACAGGTACTACGCTGTGCAGGATGTCAGTTCCGTTAAGAATGCTGAGGACATTACATCTCACGTTCCTAACTACATCCCTAATGGTGTGTTCAGTATTTGCGGAAGTGGTACGGAAAACTTCTGTTCGGTACTATCTCACGGGGACCCTAGTAAAATCTTCATg                                 <  1:303200/298‑1 (MQ=255)`  
                                                                                                                                                                                                                                                                              `cTCGGGTACTCTCACATCTAAGTCGGTTGGGTTGAACCTAACGACCCAGTTTGACGTACAGGACCGAGCGGGACCTTTTTGGATTGGGCGTAATGTCTACTTTGCTAGTCCGAGGTCCAGCTTCACGTCCATCCACAGGTACTACGCTGTGCAGGATGTCAGTTCCGTTAAGAATGCTGAGGACATTACATCACCCGTTCCTAACTACATCCCTAATGGTGTGTTCAGTATTTGCGGAAGTGGTACGGAAAACTTCTGTTCGGTACTATCTCACGGGGACCCTAGTAAAATCTTCATGTAc                              <  1:255304/301‑1 (MQ=255)`  
                                                                                                                                                                                                                                                                                       `tctcACATCTAAGTCGGTTGAGTTGAACCTAACGACCCAGTTTGACGTACAGGACCGAGCGAGACCTTTTGGGATTGGGCGTAATGTCTACTTTGCTAGTCCGAGGTCCAGCTTCACGTCCATCCACAGGAACTACGCTGTGCAGGATGTCAGTTCCGTTAAGAATGCTGAGGACATTACATCACACGTTCCTAACTACATCCCTAATGGTGTGATCAGTATTTGCGAAAGTGGTCCGGCAAACTTCTGTTCGGTACTATCTCACGGGGACCCTAGTAAAATCTTCATGTACAAATTACtt                     >  1:126751/1‑300 (MQ=255)`  
                                                                                                                                                                                                                                                                                           `acaTCTAAGTCGGTTGAGTTTAACCTAACGACCCAGTTTGACGTACAGGACCGAGCGAGACCTTTTGGGATTGGGCGTAATGTCTACTTTGCTAGTCCGAGGTCCAGCTTCACGTCCATCCACAGGTACTACGCTGTGCAGGATGTCAGTTCCGTTAAGAATGTTGAGGACATTACATCACACGTTCCTAACTACATCCCTAATGGTGTGTTCAGTATTTGCGGAAGTGGTACGGAAAACTTCTGTTCGGTACTATCTCACGGGGACCCTAGTAAAATCTTCATGTACAAATTCCTGTAcc                 <  1:237651/301‑1 (MQ=255)`  
                                                                                                                                                                                                                                                                                           `acaGCTAAGTCGGTTGATTTGAACCGAACGACCCAGTTTGACGTACAGGACCGAGCGAGACCTTTTGGGATTGGGCGTAATGTCTACTTTGCTAGTCCGAGGTCCAGCTTCACGTCCATCCACAGGTACTACGCTGTGCAGGATGTCAGTTCCGTTAAGAATGCTGAGGACATTACATCACACGTGCCTAACTACATCCCTAATGGTGTGTTCAGTATTTGCGGAAGTGGTACGGAAAACTTCTGTTCGGTACTATCTCACGGGGACCCTAGTAAAATCTTCATGTACAAATTCCTGTAcc                 <  1:65328/301‑1 (MQ=255)`  
                                                                                                                                                                                                                                                                                            `caTCTAAGTCGGTTGGGTTGAACCTAACGACCCAGTTTGACGTACAGGACCGAGCGAGACCTTTTGGGATTGGGCGTAATGTCTACTTTGCTAGTCCGAGGTCCAGCTTCACGTCCATCCACAGGTACTACGCTGTGCAGGATGTCAGTTCCGTTAAGAATGCTGAGGACATTACATCACACGTTCCTAACTACATCCCTAATGGTGTGTTCAGTATTTGCGGAAGTGGTACGGAAAACTTCTGTTCGGTACTATCTCACGGGGCCCCTAGTAAAATCTTCATTTACAAATTCCTGTACCt                >  1:490678/1‑301 (MQ=255)`  
                                                                                                                                                                                                                                                                                            `caTCTAAGTCGGTTGGGTTGAACCTAACGACCCAGTTTGACGTACAGGACCGAGCGAGACCTTTTGGGATTGGGCGTAATGTCTACTTTGCTAGTCCGAGGTCCAGCTTCACGTCCATCCACAGGTACTACGCTGTGCAGGATGTCAGTTCCGTTAAGAATGCTGAGGACATTACATCACACGTTCCTAACTACATCCCTAATGGTGTGTTCAGTATTTGCGGAAGTGGTACGGAAAACTTCTGTTCGGTACTATCTCACGGGGACCCTAGTAAAATCTTCATGTACAAATTCCTGTACCt                >  1:189820/1‑301 (MQ=255)`  
                                                                                                                                                                                                                                                                                            `caTCTAAGTCGGTTGGGTTGAACCTAACGACCCAGTTTGACGTACAGGACCGAGCGAGACCTTTTGGGATTGGGCGTAATGTCTACTTTGCTAGTCCGAGGTCCAGCTTCACGTCCATCCACAGGTACTACGCTGTGCAGGATGTCAGTTCCGTTAAGAATGCTGAGGACATTACATCACACGTTCCTAACTACATCCCTAATGGTGTGTTCAGTATTTGCGGAAGTGGTACGGAAAACTTCTGTTCGGTACTATCTCACGGGGACCCTAGTAAAATCTTCATGTACAAATTCCTGTACCt                >  1:456786/1‑301 (MQ=255)`  
                                                                                                                                                                                                                                                                                            `caTATAAGTCGGTTGAGTTGAACCTAAGGACCAAGTTTGACATACAGGACCGAGCGAGACCTTTTGGGATTGGGCGTAATGTCTACTTTGCTAGGACGAGGTCCAGCTTCACGTCCATCCACAGGTACTACGCTGTGCAGGATGTCAGTTCCGTTAAGAATGCTGAGGAAATTACATCACACGTTACTAACTACATCACTAATTGTGTGTTCAGTATTTGCGGAAGTGGTACGGACAACTTCTGTTCGGTACTATCTCACGTGGACCCTAGTAAAATATTCATGTACAAATTCCTGtccat                >  1:198373/1‑297 (MQ=255)`  
                                                                                                                                                                                                                                                                                                  `aGTCGGTTTAGTTGAACCTAACGACCCAGTTTGACGTACAGGACCGAGCGAGACCTTTTGGGATTGGGCGTAATGTCTACTTTGCTAGTCCGATGTCCAGCTTCACGTCCATCCACAGGTACTACGCTGTGCAGGATATCAGTTCCGTTAAGAATGCTGAGGACATTACATCACACGTTCCTAACTACATCCCTAATGGTGTGTTCAGTATTTGCGGAAGTGGTACGGAAAACTTCTGTTCGGTACTATCTCACGGGGACCCTAGTAAAATCTTCATGTACAAATTCCTGTACCTGAACGa          <  1:131023/301‑1 (MQ=255)`  
                                                                                                                                                                                                                                                                                                          `gagttgaACCTAACGACCCAGTTTGACGTACAGGACCGAGCGAGACCTTTTGGGATTGGGCGTAATGTCTACTTTGCTAGTCCGAGGTCCAGCTTCACGTCCATCCACAGGTACTACGCTGTGCAGGATGTCAGTTCCGTTAAGAATGCTGAGGACATTACATCACACGTTCCTAACTACATCCCTAATGGTGTGTTCAGTATTTGCGGAAGTGGTACGGAAAACTTCTGTTCGGTACTATCTCACGGGGACCCTAGTAAAATCTTCATGTACAAATTCCTGTACCTGAACGAAGAGTTaa  >  1:184723/1‑301 (MQ=255)`  

                                                                                                                                                                                                                                                                                                           `|`                                                                                                                                                                                                                                                                                                             
`CACCAACCCTTGGCCTTCTTTTGTTGGTTCAAGTATTAACGATGTGTTCTTCTTCCGTAACCGCTTAGGATTCCTTAGTGGGGAGAACATCATATTGAGTCGTACAGCCAAATACTTCAACTTCTACCCTGCGTCCATTGCGAACCTTAGTGATGACGACCCTATAGACGTAGCTGTGAGTACCAACCGAATAGCAATCCTTAAGTACGCCGTTCCGTTCTCAGAAGAGTTACTCATCTGGTCCGATGAAGCACAATTCGTCCTGACTGCCTCGGGTACTCTCACATCTAAGTCGGTTGAGTTGAACCTAACGACCCAGTTTGACGTACAGGACCGAGCGAGACCTTTTGGGATTGGGCGTAATGTCTACTTTGCTAGTCCGAGGTCCAGCTTCACGTCCATCCACAGGTACTACGCTGTGCAGGATGTCAGTTCCGTTAAGAATGCTGAGGACATTACATCACACGTTCCTAACTACATCCCTAATGGTGTGTTCAGTATTTGCGGAAGTGGTACGGAAAACTTCTGTTCGGTACTATCTCACGGGGACCCTAGTAAAATCTTCATGTACAAATTCCTGTACCTGAACGAAGAGTTAA  >  NC_001604.1/25849‑26447`

  

Alignment Legend

Aligned base mismatch/match (shaded by quality score): `ATCG/ATCG` `< 3 ≤` `ATCG/ATCG` `< 9 ≤` `ATCG/ATCG` `< 17 ≤` `ATCG/ATCG` `< 31 ≤` `ATCG/ATCG` `< 38 ≤` `ATCG/ATCG`

Unaligned base: `atcg`    Masked matching base: `atcg`    Alignment gap: `‑`    Deleted base: `‑`
